# Mobile phones as monitors of personal exposure to air pollution: Is this the future?

**DOI:** 10.1371/journal.pone.0193150

**Published:** 2018-02-23

**Authors:** Mawutorli Nyarku, Mandana Mazaheri, Rohan Jayaratne, Matthew Dunbabin, Md Mahmudur Rahman, Erik Uhde, Lidia Morawska

**Affiliations:** 1 International Laboratory for Air Quality and Health, Queensland University of Technology, Brisbane, QLD, Australia; 2 School of Electrical Engineering & Computer Science, Institute for Future Environments, Queensland University of Technology, Brisbane, QLD, Australia; 3 Material Analysis & Indoor Chemistry, Fraunhofer Institute for Wood Research, Braunschweig, Lower Saxony, Germany; Telethon Institute for Child Health Research, AUSTRALIA

## Abstract

Mobile phones have a large spectrum of applications, aiding in risk prevention and improving health and wellbeing of their owners. So far, however, they have not been used for direct assessment of personal exposure to air pollution. In this study, we comprehensively evaluated the first, and the only available, mobile phone—BROAD Life—equipped with air pollution sensors (PM_2.5_ and VOC), to answer the question whether this technology is a viable option in the quest of reducing the burden of disease to air pollution. We tested its performance, applicability and suitability for the purpose by subjecting it to varied concentrations of different types of aerosol particles (cigarette smoke, petrol exhaust and concrete dust) and formaldehyde under controlled laboratory conditions, as well as to ambient particles during field measurements. Six reference instruments were used in the study: AEROTRAK Optical Particle Counter (OPC model number 9306), DustTrak, Aerodynamic Particle Counter (APS), Scanning Mobility Particle Sizer (SMPS), Tapered Element Oscillating Microbalance (TEOM) and Formaldehyde Analyser. Overall, we found that the phone’s response was linear at higher particle number concentrations in the chamber, above 5 and 10 μg m^-3^, for combustion and concrete dust particles, respectively, and for higher formaldehyde concentrations, making it potentially suitable for applications in polluted environments. At lower ambient concentrations of particles around 10 ug m^-3^ and 20 μg m^-3^ for PM_2.5_ and PM_10_, respectively, the phone’s response was below its noise level, suggesting that it is not suitable for ambient monitoring under relatively clean urban conditions. This mobile phone has a number of limitations that may hinder its use in personal exposure and for continuous monitoring. Despite these limitations, it may be used for comparative assessments, for example when comparing outcomes of intervention measures or local impacts of air pollution sources. It should be kept in mind, however, that a mobile phone measuring air quality alone cannot as such 'reduce the burden of disease to air pollution, as knowing ambient concentrations is only one of the building block in this quest. As long as individuals cannot avoid exposure e.g. in urban areas, knowing concentrations is not sufficient to reduce potential adverse effects. Yet, there are many situations and microenvironments, which individuals could avoid knowing the concentrations and also being aware of the risk caused by exposure to them. This includes for example to proximity to vehicle emissions, either for social purposes (e.g. street cafes) or exercising (e.g. walking or jogging along busy roads)or indoor environments affected by combustion emissions (smoking, candle burning, open fire).

## Introduction

Human contact with air pollution is quantified in terms of *exposure*, which is a function of concentration of the specific pollutants and time when people are subjected to them [1]. Accurate estimation of *personal exposure* to air pollution at high spatial resolution in urban and intra-urban microenvironments is crucial in assessing the risk to individuals’ and communities from air pollution [2–4]. The need for high resolution air quality data, technological advances in high-tech device miniaturization, and the growing demand for knowledge and information on personal exposure by citizens, have a potential to shift the paradigm from conventional fixed location to mobile air pollution monitoring [3–7]. Mobile air sensing and monitoring devices with potential application in personal exposure monitoring at high temporal, and therefore high spatial resolution, are emerging [8–10]. Numerous studies utilising personal monitors to quantify and evaluate personal exposure have been reported [11–13]. The application of air sensor networks, such as wireless distributed networks of sensors to collect and disseminate real time air quality data have been designed and trailed [14–18].

Since most people carry them, the mobile phone is an obvious choice for personal exposure monitoring. However, to date, it has not been used as a stand-alone device for this purpose. There have been several approaches reported on the application of mobile phones coupled with pollutant sensing instruments to collect and disseminate air quality data [19–22]. This has been done by either directly connecting the sensing device to the mobile phone, or by interfacing it via Bluetooth pairing or tethering, for real time data visualisation on the phone and subsequent upload to a server. Hasenfratz, Saukh [22] described a study using a low-cost, low-power mobile system by interfacing HTC Hero smartphone, which runs on the Android Operating System, to a sensor that measured ozone concentrations. The micro-board was powered by a battery pack and the data transmitted using a USB-RS232 serial interface, all powered by a battery pack. Data was uploaded to the server for viewing using an Android-compatible application. Dutta et al., in 2009 demonstrated use of a portable mobile device consisting of a board, GPS receiver and an ozone sensor, combined with browser-accessible website to collect air quality data on personal scale in the Common Sense project implemented at the University of California, Berkeley. In the CitiSense project, wearable sensor boards paired with an Android phone, a server-supported web-based personalized daily pollution map, and a social component supported through Facebook and Twitter integration were deployed for a quantitative study of personal exposure among 16 participants at the University of California San Diego who used different modes of transport [23]. The study investigated human responses to the information on real time air quality and on the activities that elevate their personal exposure. The study showed that there was an improvement in understanding and awareness creation on air pollution among the participants, which affected their behaviour and attitudes, leading to lifestyle changes and better perspective of their world [20]. These findings underscore the socio-scientific benefits of mobile devices when applied for personal exposure measurements.

Despite the potential of mobile phones in application to air pollution and personal exposure measurements, to date, there has been only one type of commercially available mobile phone with in-built air pollution sensors enabling direct pollution monitoring–the BROAD Life mobile phone, which is a product of the BROAD Company in China [24]. This study aimed at: (i) comprehensive evaluation of its performance, including linearity of response, precision, detection limit and response dependence on particle size, and (ii) exploring challenges in utilization of direct monitoring of air pollution by mobile phones for personal exposure assessment.

## Materials and methods

Laboratory and field evaluation of the phone was conducted to determine comparability of the short-term output with reference instruments, applicability, and suitability for the purpose, which means whether it could be used as a tool for quantifying personal exposure to air pollution in urban environments.

### BROAD Life mobile phone LB-2

The model LB-2 BROAD Life mobile phone has dual SIM (subscriber identification module) slots, one for GSM and the other for CDMA and runs on Android OS [24]. The phone is low-power (over 12 hours battery life), battery-operated, a data-logging device that weighs approximately 200 grams and has dimensions 137 × 78 × 25 mm. Imbedded in it are six sensors used for the detection of PM, volatile organic compound (VOC), temperature, relative humidity (RH), ultraviolet (UV) radiation and electromagnetic radiation (EMR). The manufacturer’s information sheet does not provide details on the mechanism of detection of the sensors.

Two keys operate the phone: phone mode operation key and air quality detection key (S1 Fig). The phone mode has functionalities of a smartphone with features such as gallery, camera, FM radio, email, calendar, phonebook, web, etc., all of which are touch-screen operated. The air quality interface has four touch-screen operated buttons that are used for real-time detection of PM, VOC, UV and EMR. In our study, we focused on the evaluation of the phone’s PM and VOC detection capability.

The device uses a laser detection technique to count the number of particles in a known volume of air. The number concentrations are then converted to particle mass concentrations. The phone reports PM in both number and mass concentration. PM number concentration is banded into three size ranges: 0.3–2.5, 2.5–10 and >10 μm and reported in quantity per litre (Qty/L).

VOC detection is by a built-in electrochemical sensor (S1 Table), and depends on the reaction of VOC gas with an electrode to generate ions, which are measured in the form of output current. Output current flowing across an external circuit is directly proportional to the gas concentration.

Response and sample detection times for PM are 5 and 30 s, respectively and those for VOC, 10 and 90 s, respectively. During the detection process, incremental values are registered on the screen until the end of the detection. When the detection is complete, a final value for the particular measurement is recorded on-screen. Temperature and relative humidity values are recorded for every measured PM value, and stored as part of time stamps. The time against any timestamp, is the time of commencement of that particular measurement (in minutes).

The phone does not execute continuous real-time measurements, as it requires human intervention by pressing a screen button for all the operations, including measurement of every parameter. This implies that, it cannot be used for continuous measurements of any parameter without the manual pressing of buttons for every measurement.

### Instruments

Instruments of the International Laboratory for Air Quality and Health, Queensland University of Technology (ILAQH, QUT—Australia), and of the Fraunhofer Institute for Wood Research (WKI—Germany) were used as reference instruments to evaluate short term response of two BROAD phones (Model LB-2, SN 1353 and SN 1414, here after coded M1 and M2, respectively).

The PM reference instruments used in the study were an Aerosol Particle Sizer (APS, Model 3312A, SN 167; TSI Inc.), Optical Particle Counter (OPC, AEROTRAK Model 9306–02; TSI Inc.), Scanning Mobility Particle Sizer (SMPS), DustTrak II (Model DT8530 SN 3305; TSI Inc.) and Tapered Element Oscillating Microbalance (TEOM, 1405-DF; Thermo Scientific). The instruments were selected to cover a broad spectrum of PM characteristics, including number and mass concentration and number size distribution. The OPC measures only particle number concentration, while the SMPS and the APS measures particle number size distribution. From the number size distributions, making certain assumptions, it is possible to estimate the particle mass concentration. The DustTrak and TEOM measure only particle mass concentrations. Table 1 provides a summary of the operating parameters and settings of all the instruments used for PM characterisation. All the instruments used were calibrated according to procedures described in their manuals. The AL4021 continuous Formaldehyde-in-Air-and-Water-Monitor (Aero-Laser Gesellschaft für Gasanalytik mbH, Garmisch-Partenkirchen,Germany) was used to evaluate the phone’s response to formaldehyde. The system works on the principle of continuous derivatisation based on the Hantzsch reaction with fluorimetric detection. Air is continuously sampled into the device and the formaldehyde is stripped by water. In a flow reactor, the solution is mixed with acetyl acetone and ammonium acetate and heated up to 70°C. Under these conditions formaldehyde is derivatized to dihydropyridine 3,5-diacetyl-1,4-dihydrolutidine which is quantified via fluorescence spectroscopy at 412 nm. The instrument has a delay time of 5 min and a detection limit of 0.05 ppb. The instrument has high sensitivity (1 ppb) and is reliable, user-friendly and touch-screen controlled.

**Table 1:** Summary of the operating parameters and settings of all the instruments used for particle matter characterisation.

Instrument	Model	Detection mechanism	Operating size range	Bins	Sampling time	Sample flowrate	Parameters measured	Purpose for use
OPC	AEROTRAK 9306	Optical	0.3–7 μm	6	135 s	2.83 LPM	PNC	Same detection mechanism and cut-off size as the phone
APS	TSI 3312A	TOF	0.5–20 μm	50	135 s	1.0 LPM aerosol, 4 LPM sheath	PNC, PM	Adjustable settings matchable to default phone settings
EC of SMPS	TSI 3080	Electrical	20–650 nm	64	120 s scan, 15 s retrace	2 LPM aerosol, 6 LPM sheath	PNC	Fractionate particles to sizes
CPC of SMPS	TSI 3787	Light scattering	> 10 nm	64	135 s	0.6 LPM	PNC	Measure sized particles
TEOM	1405-DF	Gravimetric	< 10 μm	2 (2.5 μm, 10 μm)	6 min averaged over 1 h	3 LPM for aerosol	PM	Reports reliable mass concentrations
DustTrak II	DT8530	Optical	0.1–10 μm	1 (2.5 μm)	135 s	1.7 LPM	PM	Adjustable settings matchable to default phone settings

## Study design

The experimental procedure for the study included both laboratory and ambient measurements. In the laboratory, chamber measurements were conducted at ILAQH, QUT using three different types of particles: cigarette smoke, petrol exhaust and concrete dust, under a range of concentrations. Chamber VOC assessments were conducted at WKI, using formaldehyde. These specific types of particles and formaldehyde were chosen as they are present in urban environments, and therefore to gain an insight into the mobile phone performance when challenged with them. Ambient measurements were conducted in Brisbane, with the phones and the instruments placed at the premises of two air monitoring stations operated by the State Government.

### Particle measurements

Particles measurements were carried out in the 1 m^3^ experimental chamber. The chamber has two detachable transparent windows on its vertical sides, as well as access ports to allow for particle introduction, sampling and passing of electrical leads. The chamber is equipped with a small fan, positioned in the centre, which was used to ensure uniform air mixing. For our experiments, one of the detachable transparent windows of the chamber was removed, and the opening sealed off with a transparent polyethene sheet, large enough to allow for hand insertion into the chamber for the pressing the buttons of the phones. All unused ports on the chamber were plugged tightly during the measurements.

The chamber was thoroughly cleaned and flushed with compressed air passed through HEPA filter overnight to ensure removal of any particles from the previous measurements, and to reduce particle count to the level of the mechanically ventilated laboratory before the start of each measurement. The two BROAD phones were placed inside the chamber throughout the measurements, while the reference instruments were located outside the chamber and were drawing air from the chamber using conductive inert rubber tubes. It was ensured that the tubes were as short as possible, about 0.6 m, and it was assumed that particle loss in the tubes was insignificant and therefore had no influence on the outcomes of the measurements [25].

The PM buttons of the two phones were simultaneously pressed by extending the hand through the transparent polyethene sheet covering the chamber’s window. The buttons are touch-screen and are activated when pressed over the polyethene sheet by human finger. The sampling times of the reference instruments were set to 130–140 s so that the data points from the phones could be matched to the data from the instruments.

Before the introduction of test particles, the fan in the chamber was turned on, all instruments and phones were started simultaneously, and allowed to run for an hour to measure the chamber background concentrations. This procedure did not introduce any particles into the chamber.

#### Cigarette smoke particles

Cigarette smoke particles (3R4F, Kentucky reference cigarette) were introduced by inserting a lit cigarette into the chamber for 2 s. The measurements continued for over 3 hours until particle concentration in the chamber decreased to about 2 cm^-3^, just above the background level of 0.43 cm^-3^ (as reported by OPC).

#### Petrol exhaust particles

Petrol exhaust particles generated by a running gasoline powered vehicle, were collected by placing a Teflon bag over the nozzle of its exhaust pipe and introduced into the chamber by opening the bag in the middle of the chamber and closing the chamber window that after. The measurements were stopped after about 2 hours, when particle concentration decreased to 0.6 cm^-3^, compared to the background of 0.45 cm^-3^ (as reported by OPC).

#### Concrete dust particles

A small quantity of concrete dust was dispersed into the air of the chamber from a spatula placed in the middle of the chamber. The measurements continued for 3 hours until particle concentration in the chamber decreased to 2 cm^-3^ (background of 0.6 cm^-3^, as reported by the OPC).

#### Ambient measurements

The ambient measurements were conducted at two air quality monitoring stations, Rocklea and Woolloongabba, operated by the Department of Science Information Technology and Innovation (DSITI).

The Rocklea station is located within the grounds of the Oxley Creek Common. The environment is mainly green vegetation with light industries and residential areas at its periphery.

The Woolloongabba station is located close to the kerb of a busy main road and a commercial business area (Buranda Village). Ambient particles at the site are composed mainly of vehicular traffic emissions.

The OPC was used for the measurements, and PM_2.5_ and PM_10_ data measured by TEOM were obtained from DSITI for the measurement period. The two phones and the OPC were co-located within the precincts of the stations and the measurements continued for 3–4 hours at each station. The phones on-screen PM buttons were pressed at intervals of 35–40 s continuously. It was ensured that the measurements were carried out under weather conditions free of rain by relying on Bureau of Meteorology (BOM) forecasts.

#### VOC measurements

The phone’s response to VOC was investigated using two approaches. In the first process, the phones were exposed to a range of different VOC, including acetone, butanol, biodiesel, formaldehyde and isopropanol. The second approach was by exposing it to varying concentrations of formaldehyde alone in a chamber and comparing its readings with that of the formaldehyde analyser (AL 4021). The formaldehyde measurements were carried out using a gas chamber at WKI (S2 Fig). A fixed quantity of formaldehyde was introduced into the chamber, and its concentration was monitored using the phones and the formaldehyde analyser. The phones were enclosed in a transparent polyethene bag that was connected to the experimental chamber via a tube at one end, and the suction arm of a fume extraction system at the other end. The VOC buttons of the phones were pressed every 3–5 minutes during the measurements.

## Data analysis

Data from the phones were retrieved by manually reading and entering them into a spreadsheet. SMPS and APS concentrations were normalised by dividing raw concentration collected in each bin by the width of the bin to remove the resolution dependence and to allow for comparison of values, regardless of the channel. Normalised total particle concentration data from the APS and the SMPS, which are independent of the channel number, were processed using the AIM software. Data from the OPC were downloaded using the TrakPro Lite software. PM_2.5_ data from the DustTrak were downloaded into a USB.

The mobile phone data were aggregated into two particle size ranges: 0.3–2.5 μm and 0.3–10 μm (which are of interest to enable recalculation into PM_2.5_ and PM_10_). The OPC and the mobile phone size ranges do not correspond, and in particularly the OPC does not record particles < 2.5 μm or < 10 μm. To address this, particle concentrations from the six channels of the OPC were aggregated into two size concentration ranges of 0.3–2.1 μm and 0.3–7 μm, which are the closest to the above aggregated phone concentration size ranges.

To compare mobile phone and APS calculated PM_2.5_ concentrations of coarse particles (concrete dust); APS data were aggregated for the size range of 0.5 to 2.458 μm. The following average effective bulk particle densities were entered into the AIM software for the calculation of particle mass concentrations for the APS; cigarette smoke, 1.18 g cm^-3^, petrol exhaust, 1.20 g cm^-3^ and concrete dust, 2.20 g cm^-3^ [26, 27].

Since the detection ranges of SMPS, APS, DustTrak and phone are 20–650 nm, 0.5–20 μm, 0.1–10 μm and 0.3–10 μm respectively, PM_2.5_ concentrations of the phones were compared with DustTrak for all types of particles measured in the chamber, and with the APS for the coarse particles, concrete dust. This is because particles detectable by the SMPS have negligible mass compared with the mass of the coarser particles detectable by the phone.

TEOM PM_2.5_ and PM_10_ data obtained from DSITI were used to compare with phones’ mass concentrations for Rocklea station. TEOM data from the Woolloongabba station was not available because the TEOM malfunctioned on the day of sampling.

All instruments were time-matched to the nearest minute. Because the phones do not execute continuous monitoring and hence do not generate continuous data, data from all the instruments were synchronised to the phone's data, using time-matching the values.

## Results

### Mobile phone performance: Particle number concentration

#### Size distribution of test particles

**Fig 1:**
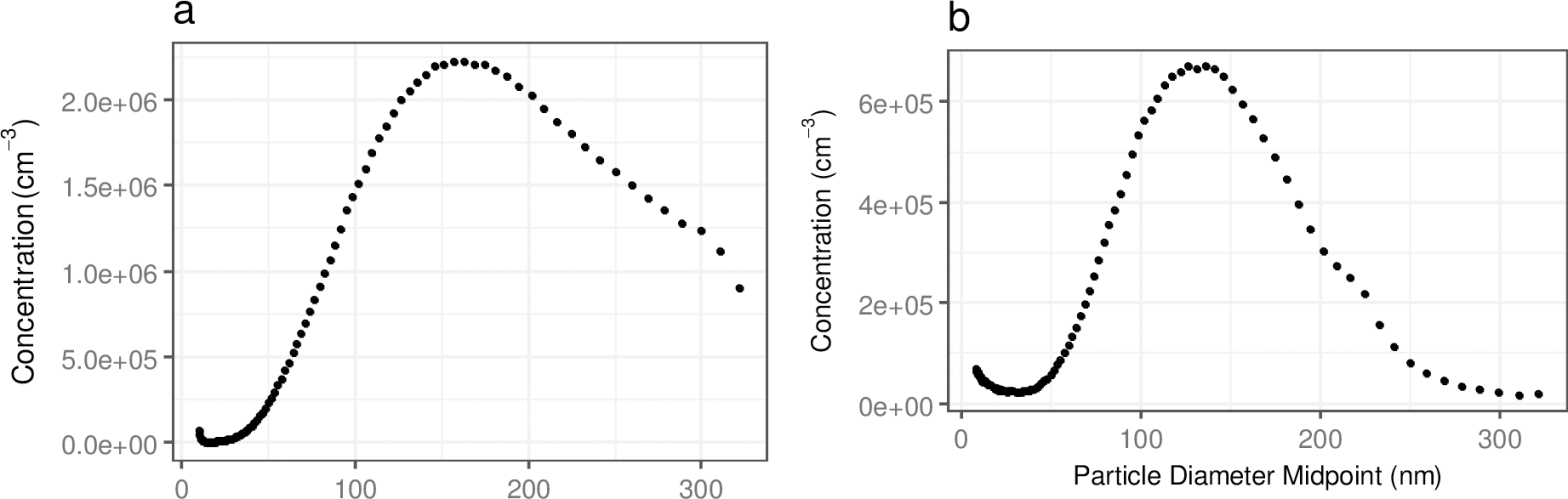
**Particle size distributions as measured by the SMPS for a) Cigarette smoke particles b) Petrol exhaust particles.** The respective number size distributions for different times were aggregated to the total size distribution.

**Fig 2:**
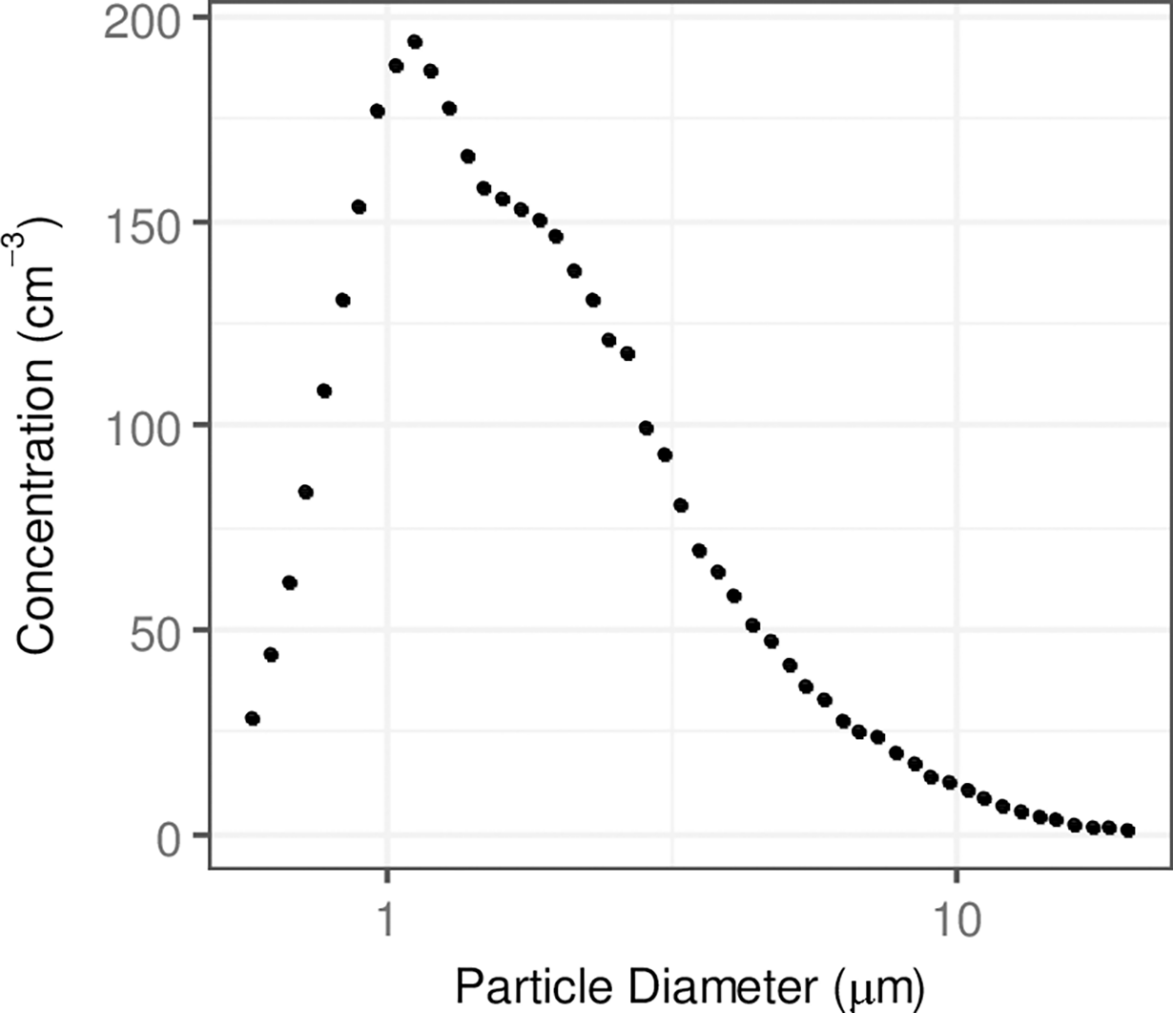
Total particle number size distribution of concrete dust particles as measured by the APS. The respective number size distributions were aggregated to the total size distribution.

Particle size distributions measured by the SMPS for the combustion particles (cigarette smoke and petrol exhaust) and by APS for concrete dust are presented in Figs 1 and 2, respectively.

It can be seen from Fig 1A that the majority of the cigarette smoke particles were < 300 nm with CMD of 160 nm, while the majority of petrol exhaust particles had sizes < 250 nm and CMD of 130 nm (Fig 1B). By contrast, the majority of concrete dust particles were < 5.0 μm with CMD of 1.2 μm.

### Measurements of particle number concentration

#### Chamber particles

**Fig 3:**
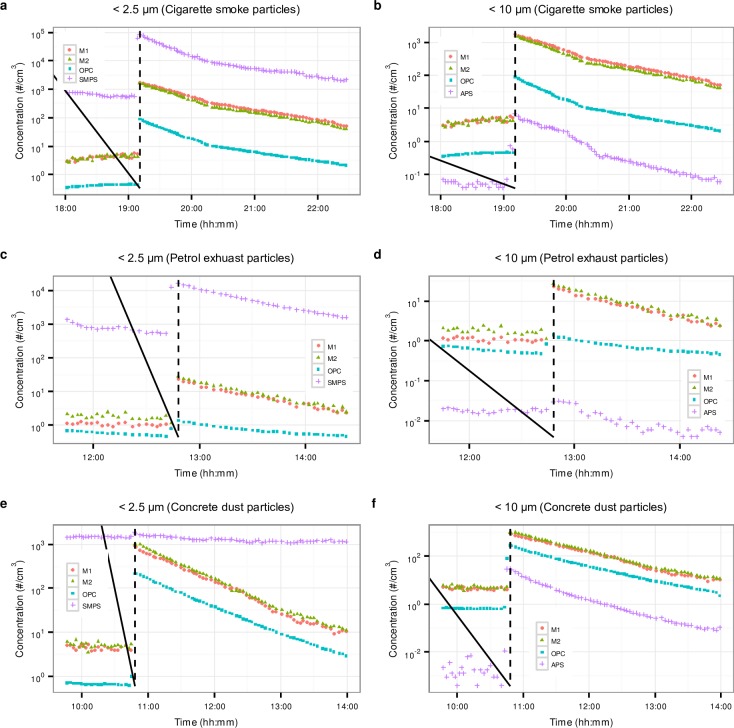
Time series of particles number concentrations measured during the chamber experiments. a) Cigarette smoke particles in the size fraction: M1 ≤ 2.5 μm, M2 ≤ 2.5 μm, OPC ≤ 2.1 μm and SMPS = total PNC. b) Cigarette smoke particles in the size fraction: M1 ≤ 10 μm, M2 ≤ 10, OPC ≤ 7 μm and APS = total PNC. c) Petrol exhaust particles in the size fraction: M1 ≤ 2.5 μm, M2 ≤ 2.5 μm, OPC ≤ 2.1 μm and SMPS = total PNC. d) Petrol exhaust particles in the size fraction: M1 ≤ 10 μm, M2 ≤ 10 μm, OPC ≤ 7 μm and APS = total PNC. e) Concrete dust particles in the size fraction: M1 ≤ 2.5 μm, M2 ≤ 2.5 μm, OPC ≤ 2.1 μm and SMPS = total PNC. f) Concrete dust particles in the size fraction: M1 ≤ 10 μm, M2 ≤ 10 μm, OPC ≤ 7 μm and APS = total PNC. The black dotted vertical lines represent times at which the particles were introduced into the chamber.

Fig 3 presents time series of particle number concentrations measured by the phones and the reference instruments for all three types of particles in the chamber.

It can be seen from Fig 3A and 3B that the OPC, SMPS, APS and the phones responded sharply to the introduction of cigarette smoke particles into the chamber by peaking to maximum concentrations. This was followed by a steady decrease in the concentrations, as expected, due to various processes taking place, in particular coagulation, deposition and removal during sampling. It can be seen that the readings of the two phones were in good agreement with each other. The phones, OPC and SMPS exhibited similar trends in response to the decreasing concentrations of the cigarette smoke particles, though there were variations between the readings of the phones and of the reference instruments, which were instrument dependent. The SMPS readings were comparatively higher because it detects particles in the ultrafine size range (<100 μm), which is the predominate size of combustion aerosols, including cigarette smoke particles [28]. For the same reason the APS readings (Fig 3B) were much lower compared to OPC and the phones, because the vast majority of the particles are smaller than the lower detection range of the instrument (≥ 0.7 μm).

In general, similar observation can be made about the response of the all the instruments and phones to petrol exhaust particles (Fig 3C and 3D). The differences are in the magnitude of variation between the readings of the various instruments and the phones and the comparative variation in readings at higher and lower particle concentrations. In particular, SMPS readings were comparatively higher in response to petrol particles than to cigarette smoke, than the responses of other instruments: this can be explained based on petrol particles being smaller than cigarette smoke particles (Fig 1). For all the particle types, the phones readings were higher than those of the OPC. It can also be seen that for the petrol exhaust particles, the variation between the readings of the phones and of the OPC changed with particle concentration (Fig 3C and 3D), with the gap between the readings decreasing with the decrease in particle concentration.

Though the SMPS readings were comparatively higher than the readings of the phones, the OPC and the APS after introduction of concrete dust into the chamber, they did not increase, and remained at the same level as without the dust (Fig 3E). This was to be expected, since the majority of the concrete dust particles are larger than 650 nm, which means that they are outside the detection range of the instrument. However, they were well within the range of the OPC and APS and both instruments showed clear decreasing trends of concentration with time. Like in the case of combustion particles, the phones showed good agreement with each other (Fig 3E and 3F) in response to the concrete dust particles. There was much less difference between the readings of the phones and of the OPC in response to concrete dust than to combustion particles; this is because the particles are within the detection range of both, the phones and OPC, and the detection ranges are similar.

#### Ambient particles

**Fig 4:**
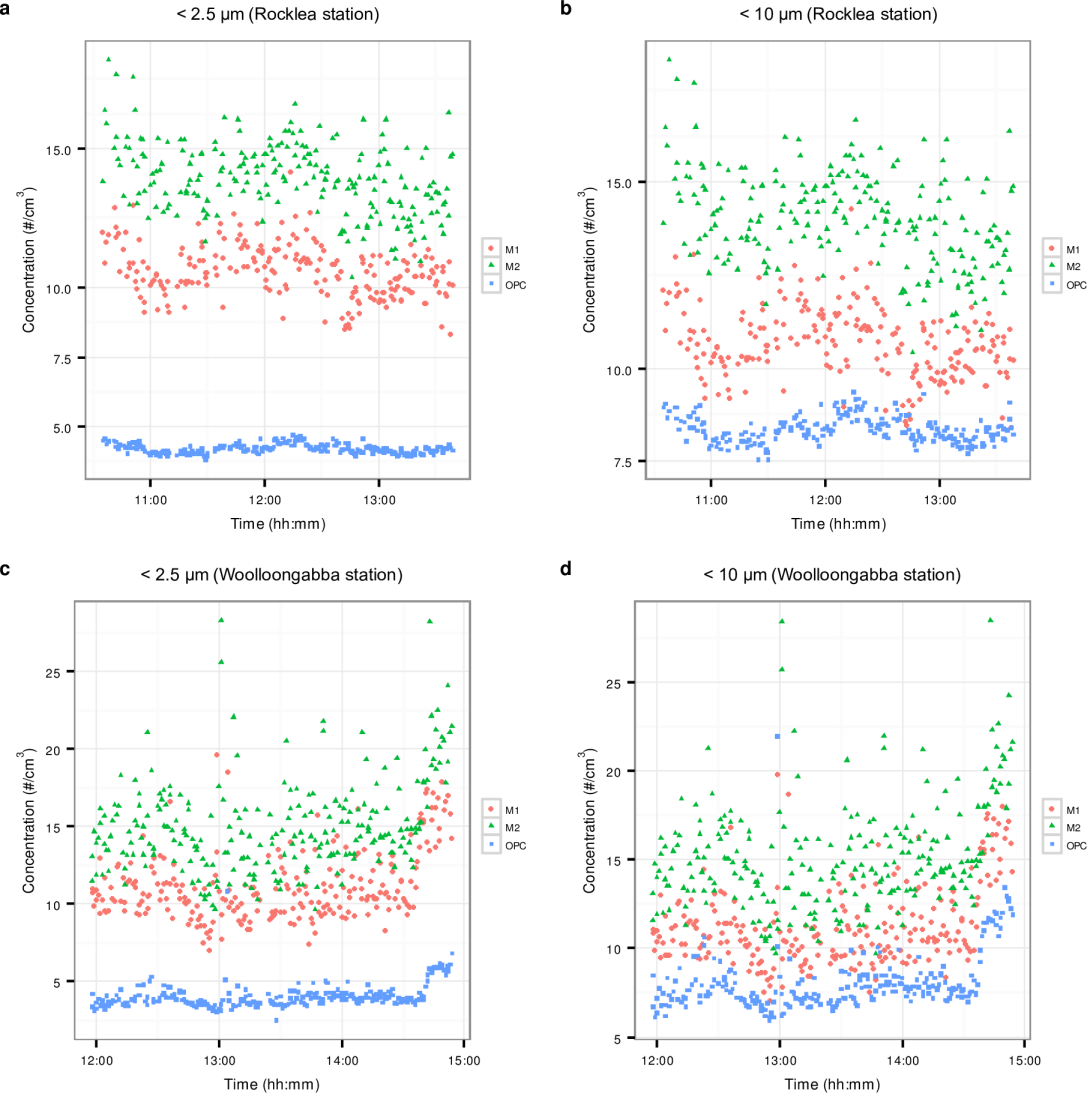
Time series of ambient particles number concentrations. a) Rocklea station, particles in the size range: M1 ≤ 2.5 μm, M2 ≤ 2.5 μm and OPC ≤ 2.1 μm. b) Rocklea station, particles in the size range: M1 ≤ 10 μm, M2 ≤ 10 μm and OPC ≤ 7 μm. c) Woolloongabba station, particles in the size range: M1 ≤ 2.5 μm, M2 ≤ 2.5 μm and OPC ≤ 2.1 μm. d) Woolloongabba station, particles in the size range: M1 ≤ 10 μm, M2 ≤ 10 μm and OPC ≤ 7 μm.

Fig 4 presents time series of the ambient particle number concentrations measured by OPC and the phones at the Rocklea and Woolloongabba stations.

Inspection of Fig 4 reveals that, as reported by the OPC and the phones, particle concentrations were in general higher at the Woolloongabba than the Rocklea station, which was as expected, since the former is influenced by close proximity of exhaust and no-exhaust traffic emissions. In both cases, the phone’s readings were significantly higher than those of the OPC, which cannot be explained by the small differences in the size windows into which the readings were aggregated. Further, the scatter of the phones’ responses were significantly higher than that of the OPC, and there was a difference in readings between the phones, with M1 reading less than M2. It can be concluded that at these particle concentration levels, which are typical of ambient air in Brisbane, the phone’s response is very noisy.

#### Correlation between the responses of the phones and the OPC

To compare variation in response of the phones and of the OPC to changing particle concentrations, their coefficient of determination (R^2^) values were calculated. This was done for concentrations of particles < 2.5 μm (phones) and < 2.1 μm (OPC), since this size is of a particular interest in ambient monitoring, as it is relevant to PM_2.5_. High R^2^ values were found between the phones and the OPC (0.85 ≤ R^2^ ≤ 1.00) for all particle types measured in the chamber. However, under ambient conditions, the R^2^ values between the phones and the OPC for M1 and M2 were respectively 0.10 and 0.23 for Rocklea; and 0.28 and 0.15 for Woollloogabba stations. It is instructive to note that the concentrations in the chamber were much higher than the typical ambient concentrations in Brisbane. This points out to the utility of the phones for measurements in environments where concentrations are high, but not for ambient monitoring.

### Mass concentrations

#### Time series plots

**Fig 5:**
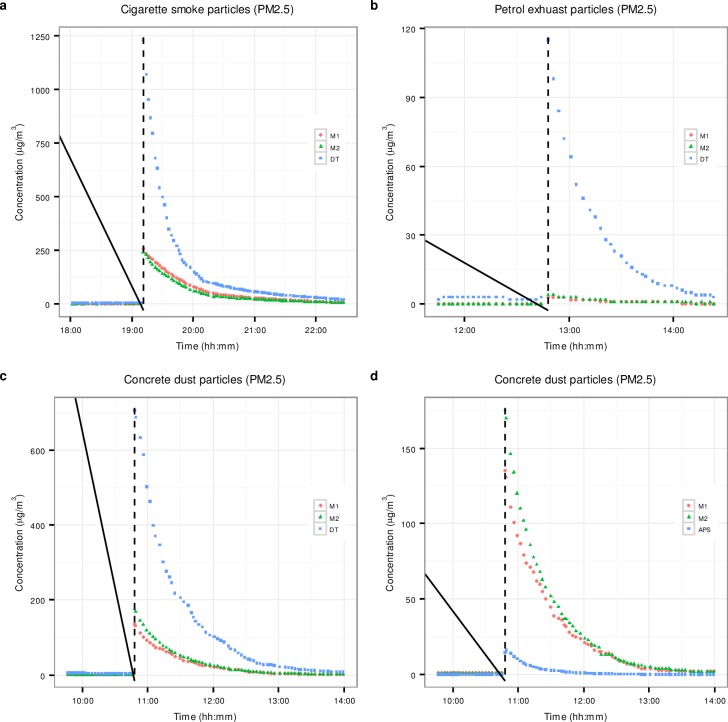
Time series of PM_2.5_ concentrations measured during chamber experiments. a) Cigarette smoke particles measured by M1, M2 and DustTrak. b) Petrol exhaust particles measured by M1, M2 and DustTrak. c) Concrete dust particles measured by M1, M2 and DustTrak. d) Concrete dust particles measured by M1, M2 and APS. The black dotted vertical lines indicate the times at which the particles were introduced into the chamber.

Fig 5 presents time series of PM_2.5_ concentrations measured by the phones and the DustTrak for all three particle types measured in the chamber, and by the phones and the APS, for concrete dust particles.

Fig 5 shows that before the introduction of cigarette smoke into the chamber, the responses of the phones and the DustTrak were comparable. However, immediately after introduction of the smoke, the DustTrak readings were several times higher than the phones—around 1250 μg/m^3^, as opposed to 250 μg/m^3^. This is not surprising: as explained above, only the larger cigarette smoke particles are detected by the phones. With time, the readings of the DustTrak and the phones became comparable again, when the concentration of the particles decreased and their count median diameter increased because of coagulation and faster diffusional deposition of the smaller than the larger particles.

The introduction of petrol smoke into the chamber resulted in a sharp response of the DustTrak, similar to its response to cigarette smoke, however, by contrast to cigarette smoke, there was no response from the phones. Again, it is not surprising, considering that petrol exhaust particles are even smaller than the cigarette smoke particles, and not detectable by the phones.

The phones, DustTrak and APS all reported low and comparable concentrations before the introduction of concrete dust particles, and all the instruments responded sharply to the introduction of the dust. However, there was a significant difference in the magnitude of the responses, with the maximum concentrations, reported by the DustTrak, APS and the phones, being 900 μg/m^3^, 16 μg/m^3^ and 180 μg/m^3^, respectively. The APS has a relatively high lower particle size detection level of 0.7 μm. This may partially explain why its readings were much lower than the other two instruments.

**Fig 6:**
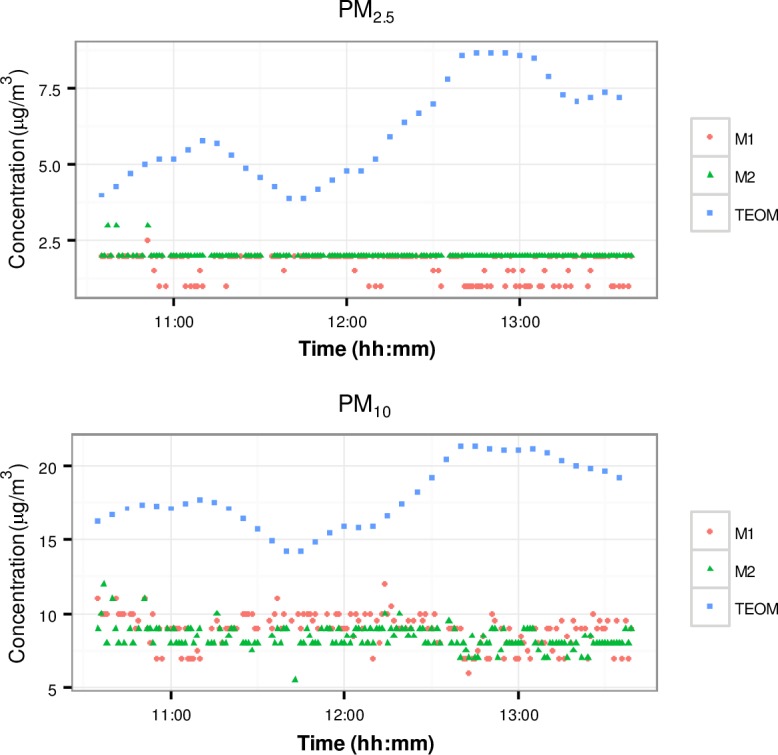
Time series of mass concentrations at the Rocklea station measured by the phones and the TEOM.

Fig 6 presents time series of ambient PM_2.5_ and PM_10_ concentrations measured by the two mobile phones and the TEOM at the Rocklea station. No data were available for the Woolloongabba station because the TEOM monitor was unexpectedly out of service during the period of the measurement.

It can be seen from Fig 6 that the concentrations reported by the two phones did not match those of the TEOM, neither for PM_2.5_ nor for PM_10_, and in fact did not vary with the concentrations. It can be concluded that at these relatively low ambient concentrations, the phone’s output is noise (as seen in Fig 3D).

#### Correlation between the phones and the DustTrak

We compared the variations in response to changing particle concentrations of the phones and of the DustTrak, by calculating their coefficient of determination (R^2^) values for the chamber measurements. For cigarette smoke, the R^2^ values were 0.90 for M1 and 0.94 for M2, and for concrete dust, they were 0.99 for M1 and 1.00 for M2. For petrol exhaust, the R^2^ values were 0.86 for M1 and 0.87 for M2. There is a strong relationship between the readings of the mobile phones and the DustTrak, in terms of their response to varying concentration of particles, similar to what was observed for the phones and the OPC in the chamber measurements.

### VOC measurements

**Fig 7:**
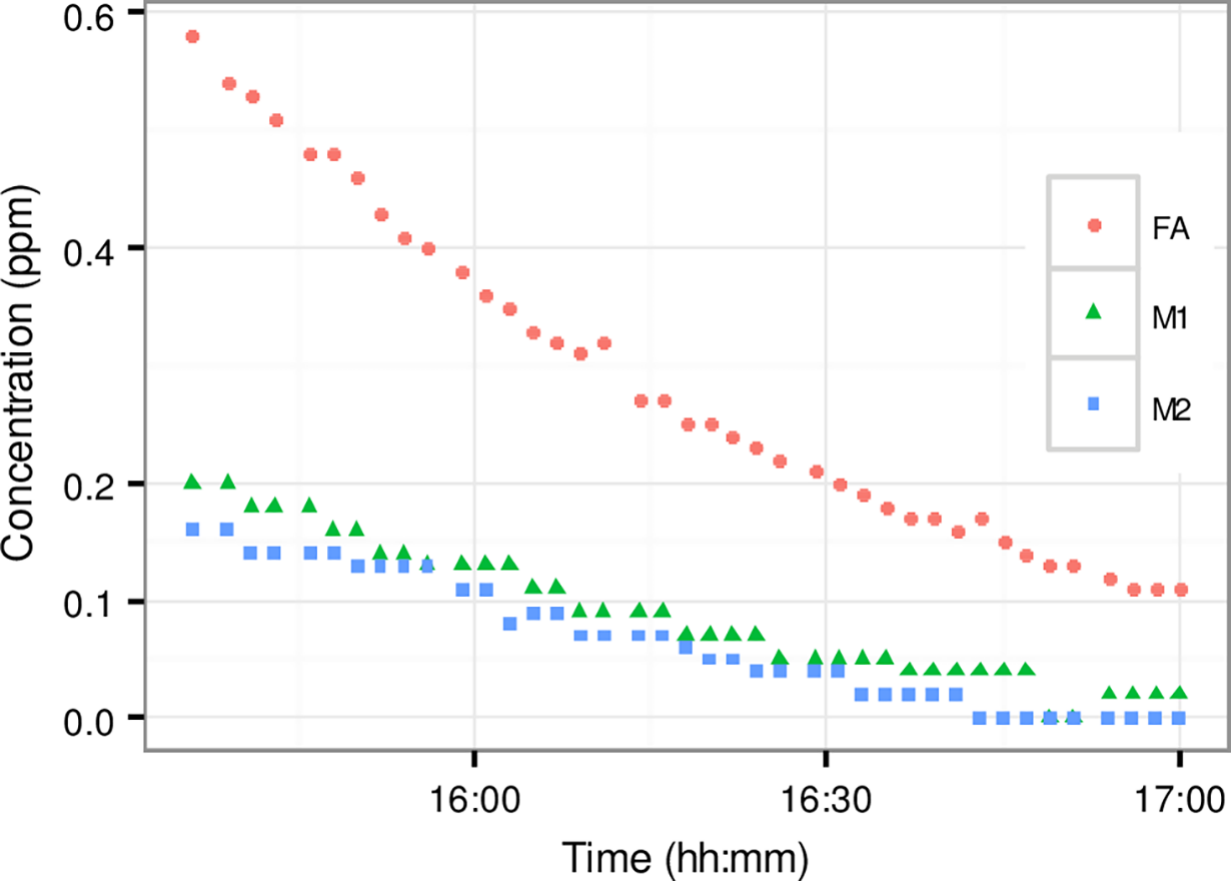
Response of phones (M1 and M2) and formaldehyde analyser (FA) to varying concentrations of formaldehyde in chamber.

Fig 7 presents time series of the responses of the phones and formaldehyde analyzer to varying concentrations of formaldehyde.

As can be seen from Fig 7, the phones underestimate the formaldehyde concentrations and have low resolution in response to formaldehyde, indicated by the discrete horizontal steps of the values in the graph. However, there is a good correlation between the phones and the formaldehyde analyser, with R^2^ of 0.98 and 0.97 for M1 and M2, respectively.

Table 2 presents a summary of the results obtained from testing the phones’ response to different VOC sources. As can be seen from Table 2, the response varied, dependently on the VOC source, but there was no response to dish washing liquid and laundry detergent. The measurement range of the phone for VOC is 0–3. The response was categorized according to the following scale: strong (readings > 2.5 ppm), medium (2.5 ppm < readings > 0.1 ppm), weak (readings < 0.1 ppm) and none (no response).

**Table 2:** Response of the phones to the VOC sources. Strong (readings > 2.5 ppm), medium (2.5 ppm < readings > 0.1 ppm), weak (readings < 0.1 ppm) and none (no response).

Source	Response			
	Strong	Medium	Weak	None
***Chemicals***				
Acetone	×			
Butanol	×			
Ethanol	×			
Formaldehyde		×		
Isopropanol	×			
Biodiesel		×		
Lime Juice (Citric acid)			×	
Dish washing liquid				×
Laundry detergent				×
Vinegar (Acetic acid)		×		
***Vehicle emissions***				
CNG fueled bus			×	
Petrol	×			
Diesel		×		
LPG			×	

## Discussion and conclusions

The first and the only so far mobile phone equipped with sensors for direct measurements of selected air pollutants (PM_2.5_ and VOC), BROAD Life, was comprehensively tested and evaluated. The main question was whether it could be used confidently and reliably as a tool for individuals to monitor their personal exposure as they move between various outdoor and indoor microenvironments.

The phone was exposed in laboratory chamber experiments to two types of common combustion type particles (petrol emissions and cigarette smoke), as well as concrete dust. During field experiments, it was exposed to ambient particulate matter, at relatively low concentrations, typically found in urban air in Brisbane.

We showed that the responses of the two phones were in good agreement with each other for all chamber experiments, both for particles and formaldehyde. However, there were some exceptions as seen in S2 and S3 Figs, where the readings of the two phones did not correspond well to each other. We cannot explain this difference by the positioning of the phones in the chamber. It is probably a feature of the variability of the built-in low-cost particle counters. At higher particle concentrations, above 10 cm^-3^ and 50 cm^-3^ (equivalent to 5 μg m^-3^ and 10 μg m^-3^ for PM_2.5_) for combustion and concrete dust particles, respectively, there was a good liner correlation between the readings of the phone and the reference instruments. Correlation coefficients (R^2^) relating the readings of the phones and the individual instruments were as follows: OPC (0.85 ≤ R ≤ 1.00), DustTrak (0.86 ≤ R^2^ ≤ 1.00) and Formaldehyde Analyser (0.97 ≤ R ≤ 0.98). Performance of the phone is particle type dependent and of all the particle types tested, concrete dust particles gave the best correlations, of very close to 1.00. This is not surprising, considering that all the concrete dust particles are within the detection range of the optical sensor employed by the phone. This is unlike the combustion particles, with the majority of them smaller than the sensor’s lower detection limit of 0.3 μm at which the counting efficiency of OPC and the phone is only 50%. Unfortunately, at lower ambient particle concentrations, at the level of 10 μg m^-3^ for PM_2.5_ and 20 μg m^-3^ for PM_10_, typical to outdoor air in Brisbane, the phones’ response was noisy, making them unusable under such conditions. In conclusion, the phone’s linear response under higher particle concentrations makes it potentially suitable for applications in polluted environments, but not suitable for ambient monitoring under relatively clean urban conditions.

While the response of the phones was linear at high concentrations with all three types of aerosols used, it differed from the response of the reference instruments by orders of magnitude. This was also the case for the OPC, which has the same cut-off as the phones.

Out of the particle counting reference instruments used, it was confirmed that the OPC was the most appropriate instrument to compare the phone with, because of their common lower detection particle size limit of 0.3 μm. However, availability of particle size distributions of the test aerosols provided by the SMPS and APS, as well particle mass concentration (DustTrak), provided additional and more in depth insights, which enabled better interpretation of the results.

We were not able to compare the response of the BROAD Life phone to other phones, as there are no other such phones on the market. However, we have compared its response to the performance of other sensors challenged with different particle types as described in literature. The response of the phone to particles depends on the specifications of the sensor that is employed by the phone. In most of the previous studies evaluating performance of low-cost sensors, test particles were utilised under laboratory conditions [29] tested the Shinyei PD42NS particle sensor in an exposure chamber using monodisperse polystyrene spheres of diameters of 0.75, 1, 2, 3 and 6 μm and ASHRAE test dust of sizes 0.5–20 μm, and found that, the sensor’s response was linear for all particle sizes tested at concentrations up to 100 μg/m^3^. Since the sensor detects particles > 0.5 μm, the authors pointed out that it is not suitable for assessing exposures to ultrafine particles. Wang et al [30] evaluated three low-cost optical sensors based on calibration methods developed by the US EPA. SMPS, SidePak and AirAssure, were the reference instruments used to test six performance aspects of the sensors. All three sensors displayed linear responses to the particle concentrations as demonstrated against the reference instruments. For instance, for NaCl particles, two of the sensors (PPD and GP2Y) exhibited linear responses in the concentration range of 0–500 μg/m^3^. For sucrose, the PPD, GP2Y and DSM exhibited linear responses in the concentration ranges 0–100, 0–150 and 0–50 μg/m^3^, respectively. It was also found that the readings of the sensors depended very much on particle composition and size, with the differences between the sensor and reference instruments readings varying by orders of magnitude. Holstius, Pillarisetti [8], conducted investigations on Shinyei PPN42SN sensors using the Federal Equivalent Method (FEM) β-attenuation monitor and two other reference instruments to calibrate the sensors by comparing and analysing the outputs of hourly and 24 hour averaged data. For usability, the naked Shinyei PPN42SN sensor was used to build a custom-made battery-operated monitor, which was co-located with US EPA standard instruments at West Oakland regulatory monitoring station. While the study showed many prospects for the use of the sensors for real-time spatiotemporal community exposure assessment, they also reported inter-device variability. For example, there were variations up to 60% and 72% in the hourly and 24 h data, between the reference instruments and the sensor for ambient concentrations < 20 μg/m^3^. In summary, comparing the results of the phone evaluation in this study, with sensor evaluations reported in the literature, we conclude that the results broadly agree in terms of linearity at higher concentrations of the test particles utilized, and also regarding limited applicability of the phone, similarly to the sensors, under typical urban concentrations of real ambient aerosols.

However, the phone has several intrinsic deficiencies, especially in applications relating to personal exposure monitoring. Firstly, the phone does not measure continuously, but conducts a single measurement on a press of a button. Without the ability to measure continuously, the phone cannot be used for personal exposure assessment. One reason why it does not measure continuously is that this would have a big toll on the battery and drain it very fast. Again, this could possibly be solved with future much better batteries, but this is not quite in sight yet. Another deficiency in using this phone for personal exposure measurements is that there is the possibility of its inlet being obstructed. If the inlet is partially obstructed, the flow rate will not match the specified value, leading to inaccurate particle concentration readings. Further, the pump drawing in air into the phone could be damaged when the inlet is blocked. For this reason the phone cannot measure continuously (short of the user switching on and off the measurement when taking it out of or putting into the pocket, which would not be practical), and therefore the application of the phone to personal exposure monitoring is very limited.

There are also some other technical inconveniences limiting the BROAD Life phone’s use for personal exposure monitoring. They include the difficulty in retrieving data from the phone (they cannot be downloaded but only read), which could be easily solved, as well as the noise during the measurement, which would be a source of inconvenience to its user; this means that in the future a silent pumps would be need to be considered.

However, although it is not suitable for personal exposure monitoring, the phone can be used for checking the instantaneous levels of the pollutant concentrations. The value of this would not be in numerically relating them to the risk, via the WHO guidelines, because for PM_2.5_ the guidelines are expressed as 24 h and annual averages. It would be, however, in raising community awareness of air quality, and in educating citizens about differences between pollutant concentrations in clean and polluted environments. Yet, there would be a high price to pay for this, as phones equipped with sensor are much more expensive than standard phones.

In professional applications, where compliance monitoring of exposure is required, the quality of the phone sensors is not adequate. However, they could have applications as alarm devices for very high concentrations, for example where spot-checking is of interest, for quick detection of source emissions or identification of spatial variation and concentration gradients, and where accuracy of the measurement is not important. In such cases, the phone would have an application as a de facto research instrument. Whether this would be of sufficient interest, is not clear. With the emergence of low-cost sensors and their packages, the phone may not be an instrument of choice to monitor pollutant concentrations.

Future application of the phone for personal exposure assessment will more likely be related to using it as a location tracker (via its GPS), and connecting this information via appropriate apps with live ambient air pollution maps, of good spatial distribution, to calculate real time and cumulative personal exposure. This approach, while practical and feasible, will not provide information on exposures indoors, as it will be entirely based on the outdoor pollution monitoring. To make it possible, however, the phone would need to be equipped with a sensor sensitive to the entire pollutant concentration range encountered in indoor environments, thus encompassing also low concentrations. This is where the phone would be useful.

## Supporting information

S1 Table Technical characteristics of the phone according to the manufacturer's data sheets [24]. (DOCX)

S1 Fig Photograph showing BROAD Life mobile phone interfaces. The left photo shows the smart phone mode and the right photo shows the air quality mode.
(TIF)

S2 Fig Experimental setup to investigate the mobile phones’ response to formaldehyde. (TIF)

S3 Fig Scatter plots of the phones versus OPC for particles of sizes 2.5 μm for the phones, and 2.1 μm for the OPC. (TIF)

S4 Fig Scatter plot of PM2.5 concentrations of phones versus DustTrak showing linear relationship between the phones and the DustTrak for cigarette smoke particles. (TIF)

S5 Fig Scatter plot of formaldehyde analyzer and mobile phones. (TIF)
