# Supplementary material for: Mobile phones as monitors of personal exposure to air pollution: Is this the future?
Source: PLoS One. 2018 Feb 23;13(2):e0193150. doi: 10.1371/journal.pone.0193150 (PMC5825064; doi:10.1371/journal.pone.0193150)
Supplement: S1 Table — (DOCX) [file pone.0193150.s001.docx]

**S1 Table. Technical characteristics of the phone according to the manufacturer's data sheets [24]**

| **Sensor name** | **PM Sensor** | **VOC Sensor** | **EM Radiation Sensor** | **UV Sensor** | **Temperature Sensor** | **Humidity Sensor** |
| --- | --- | --- | --- | --- | --- | --- |
| Key function | Detects PM | Detects formaldehyde, toluene, acetaldehyde and other VOC | Detects electromagnetic radiation | Detects ultraviolet sunlight intensity | Detects ambient temperature | Detects ambient humidity |
| Detection technique | Laser + suction pump | Electrochemical | Electron | Light sensing | Electron | Electron |
| Unit | pcs/L and µg/m^3^ | ppm | µW | Level 0-15 | ^o^C | % |
| Detection time | 30 s | 10 s | Immediately | Immediately | Immediately | Immediately |
| Tolerance | ±10% | ±5% | ±10% | ±7% | ±1% | ±5% |
| Detection lifetime | 1500-20000 depending on pollution level | Unlimited | Unlimited | Unlimited | Unlimited | Unlimited |
| Maintenance | Cleaning, calibration or replacement | Calibration or replacement every 2 years | Calibration | Calibration | Calibration | Calibration |
