# Supplementary figures and images for: Mobile phones as monitors of personal exposure to air pollution: Is this the future?
Source: PLoS One. 2018 Feb 23;13(2):e0193150. doi: 10.1371/journal.pone.0193150 (PMC5825064; doi:10.1371/journal.pone.0193150)

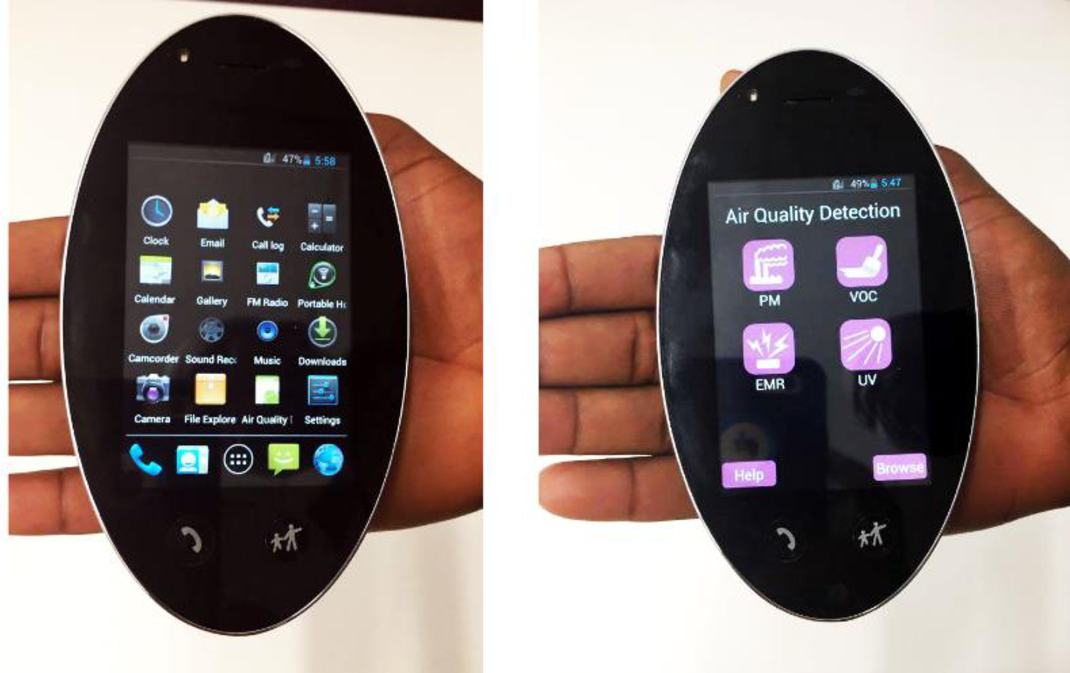

Supplement: S1 Fig — The left photo shows the smart phone mode and the right photo shows the air quality mode. (TIF) [file pone.0193150.s002.tif]

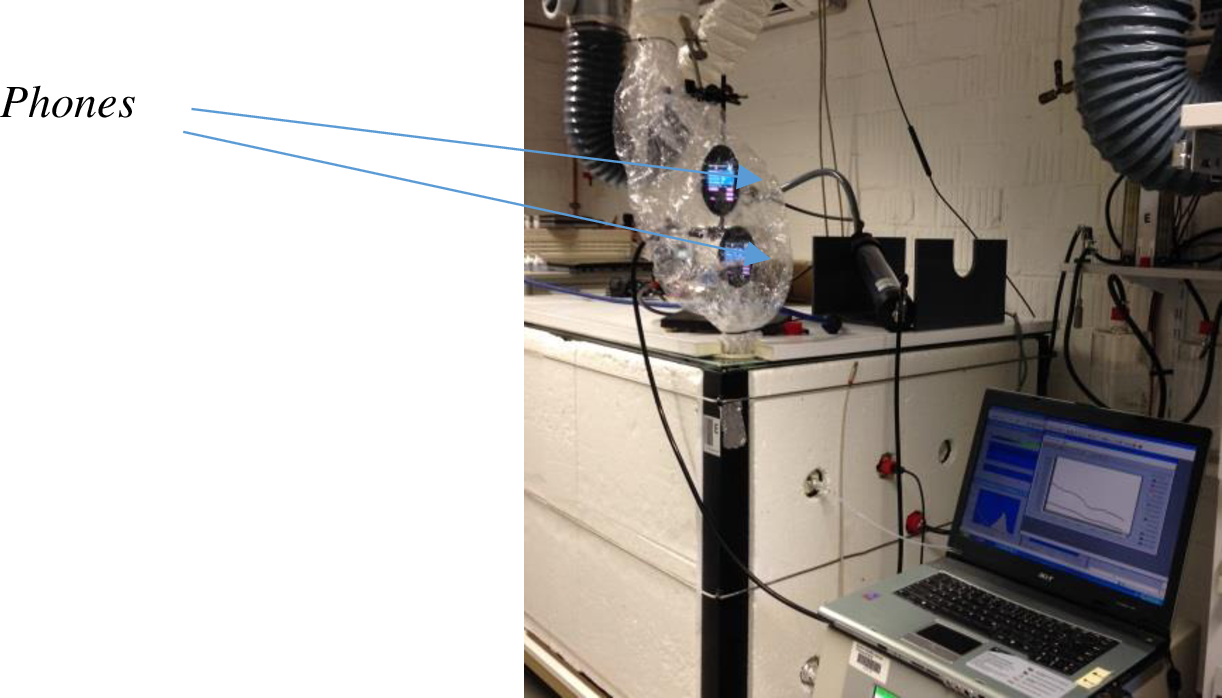

Supplement: S2 Fig — (TIF) [file pone.0193150.s003.tif]

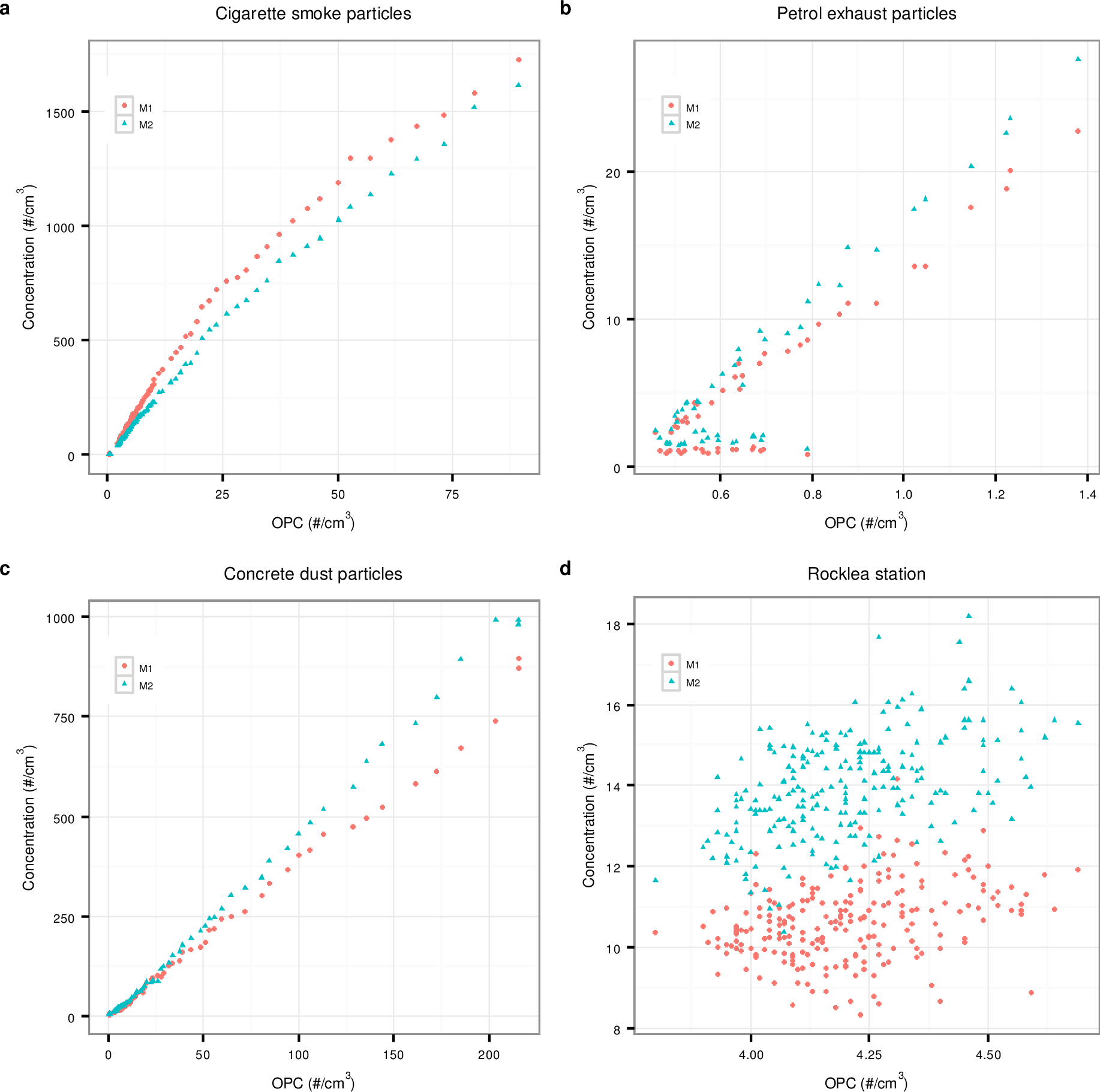

Supplement: S3 Fig — (TIF) [file pone.0193150.s004.tif]

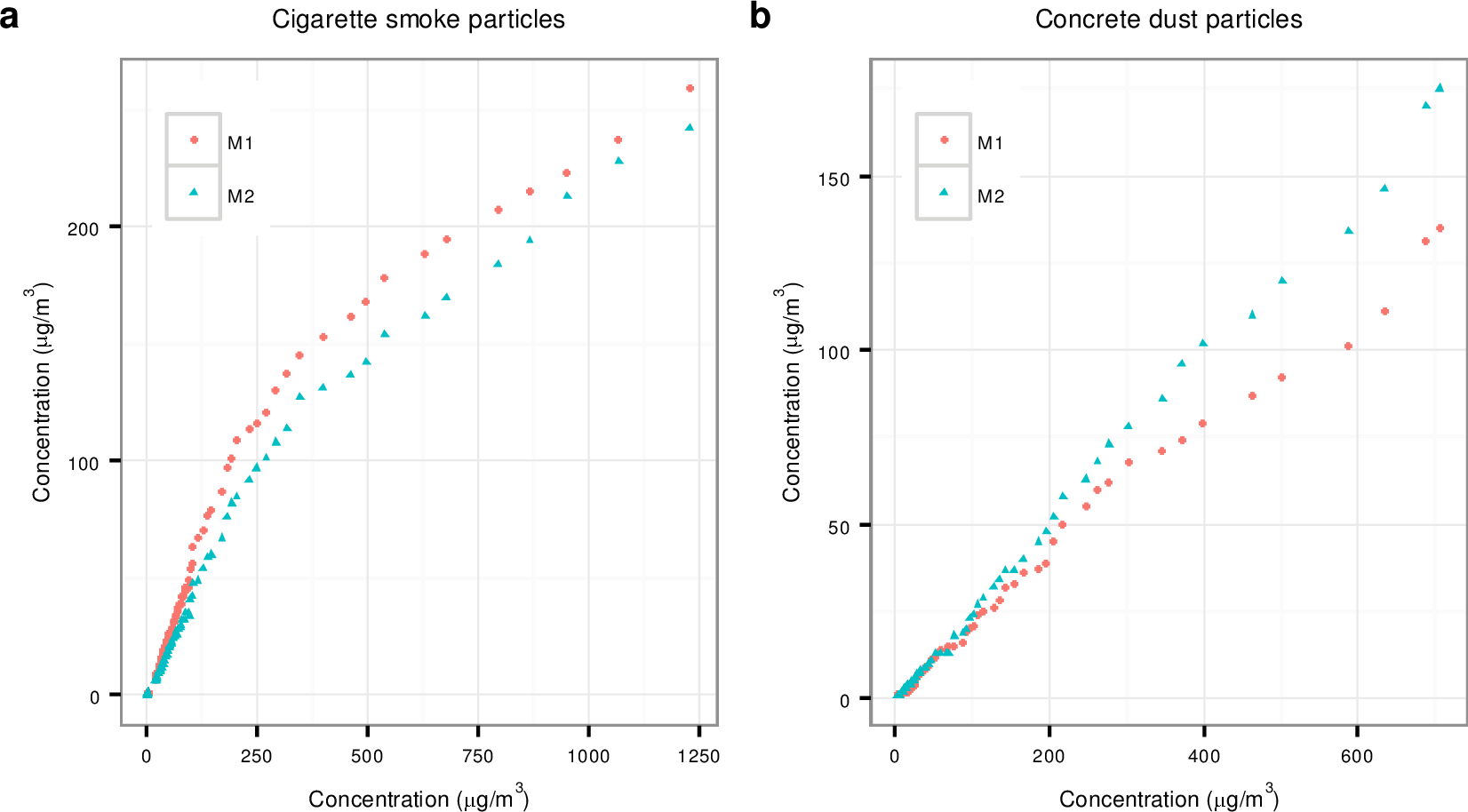

Supplement: S4 Fig — (TIF) [file pone.0193150.s005.tif]

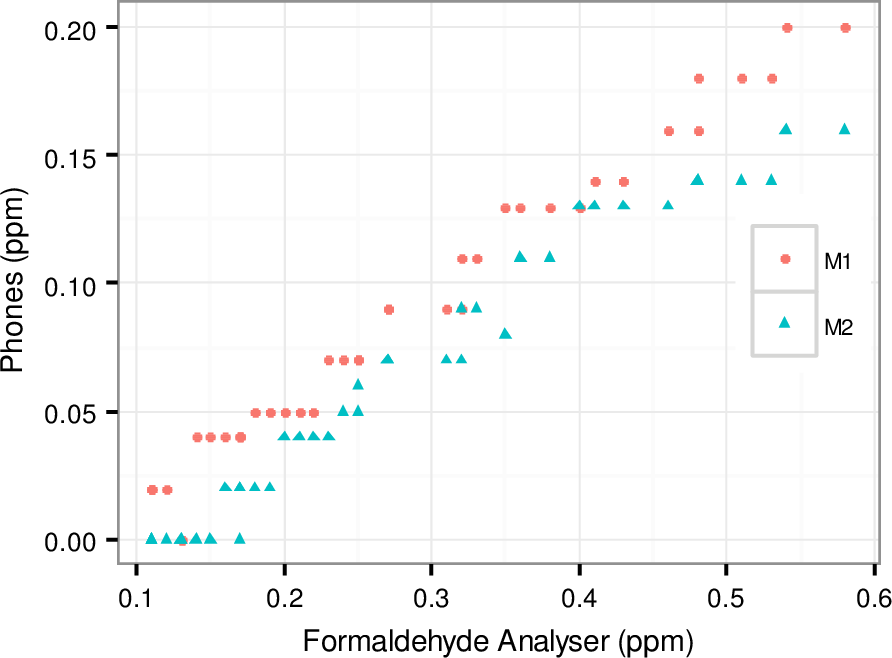

Supplement: S5 Fig — (TIF) [file pone.0193150.s006.tif]
